# Subwavelength Fabry-Perot resonator: a pair of quantum dots incorporated with gold nanorod

**DOI:** 10.1186/1556-276X-7-546

**Published:** 2012-10-02

**Authors:** Jiunn-Woei Liaw, Chun-Hui Huang, Bae-Renn Chen, Mao-Kuen Kuo

**Affiliations:** 1 Department of Mechanical Engineering, Chang Gung University, 259 Wen-Hwa 1st Rd, Kwei-Shan, Tao-Yuan, 333, Taiwan; 2 Center for Biomedical Engineering, Chang Gung University, Tao-Yuan, 333, Taiwan; 3 Institute of Applied Mechanics, National Taiwan University, 1 Sec. 4, Roosevelt Road, Taipei, 106, Taiwan

**Keywords:** Gold nanorod, Quantum dot, Longitudinal plasmon mode, Fabry-Perot resonator, Radiative power, Nonradiative power, Gold nanowire, Bi-dipole

## Abstract

The two apexes of an elongated gold nanorod (GNR) irradiated by a plane wave are shown to be the hotspots at the longitudinal plasmon modes. This phenomenon implies that a pair of quantum dots (QDs) located at these apexes might be excited simultaneously if the excitation band of QDs coincides with one of these modes. Consequently, a coherent emission of the two emitters could happen subsequently. In the following coherent emission, these two-level emitters are simulated as two oscillating dipoles (bi-dipole) with some possible phase differences. Our results show that the maximum radiative and nonradiative powers of the bi-dipole occur at the longitudinal plasmon dipole, quadrupole, sextupole, and octupole modes of GNR. Moreover, the strongest emissions are induced by the in-phase bi-dipole coupled to the odd modes and the 180° out-of-phase one to the even modes, respectively. The excitation and emission behaviors of a pair of QDs incorporated with GNR demonstrate the possibility of using this structure as a subwavelength resonator of Fabry-Perot type. In addition, the correlation between these modes of the GNR and the dispersion relation of gold nanowire is also discussed.

## Background

Single photon of a quantum dot (QD) coupling with the surface plasmon polaritons of metallic nanowire has attracted wide attentions recently [1-4]. In addition, the dispersion relations of the surface plasmon polaritons (or waves) along gold or silver nanowire [5-9] and the longitudinal plasmon modes of gold or silver nanorods [10,11] have been studied extensively. The nanoantenna effect and Fabry-Perot resonator of gold nanorod (GNR) through the longitudinal plasmon modes for the emission of nanoemitters (e.g., QD and molecule) have also been studied in the past decade [12-14]. The correlation between the surface plasmon polaritons of metallic nanowire and the plasmon modes of nanorod is an important pivot in linking the submicron and the nano-optics [15,16]. Because the lower-order plasmon modes of an elongated metallic nanorod are within the near-infrared (NIR) regime [14], it is particularly worth for study. Recently, these longitudinal plasmon modes of nanorods and nanowires have been investigated using the electric energy loss spectroscopy (EELS) [17-20]. Moreover, the plasmon-enhanced fluorescence of a fluorophore end-linked to GNR has also been demonstrated [21]. In addition, the exciton-plasmon structure of two identical QDs coupling to gold nanoparticle has been studied theoretically [22].

In this paper, the longitudinal plasmon modes of an elongated GNR irradiated by a plane wave will be studied first to illustrate that the apexes of GNR are the hotspots at these modes. This phenomenon implies that a pair of QDs at these areas might be excited simultaneously with the aid of the plasmon modes of GNR. Once the two QDs are excited and start to emit photon coherently, they are modeled as two electric dipoles with a phase difference in our analysis. To clarify the transition roles from metallic nanorod to nanowire, we investigate the plasmonic enhancement of an elongated GNR with a higher aspect ratio (AR), e.g., AR = 8, on the luminescence of nearby QDs. The far-field radiation patterns and the near-field distributions of the system will be analyzed, particularly at longitudinal plasmon modes of the GNR. In addition, the correlation between these modes and the dispersion relation of a gold nanowire (GNW) will be addressed.

## Methods

**Figure 1:**
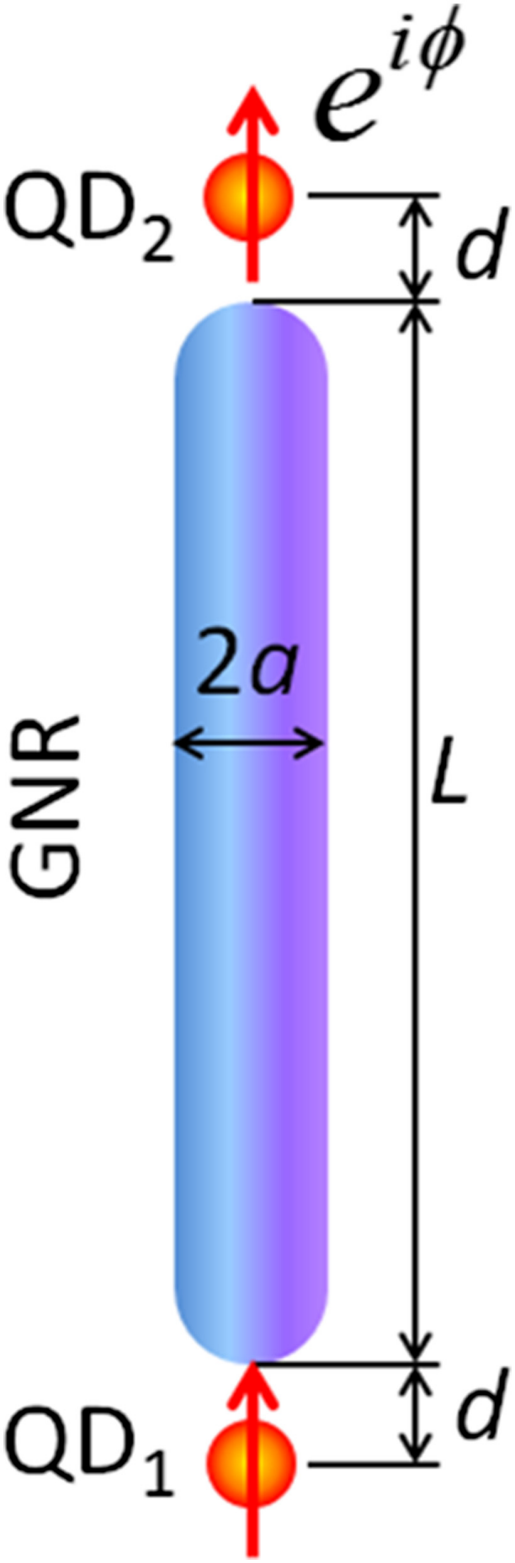
**Configuration of a pair of emitting QDs incorporated with GNR; where QDs are modeled as bi-dipole with phase difference *φ*.**

In this paper, we study theoretically the emission of two QDs located, respectively, at the two ends of an elongated GNR, as shown in Figure 1. These two QDs are modeled as two identical electric-dipole emitters, oscillating with some possible phase differences. The problem is dealt with classical electromagnetic theory. The QDs are assumed to align along the central line of GNR, where the distance between QDs and GNR is denoted by *d*. The geometry of GNR is assumed to consist of a circular cylinder with two hemispherical end-caps. The radius of GNR is denoted by *a*, and the length is by *L*. The AR of GNR is defined as *L*/(2*a*). The orientations of the dipole moments of the two QDs are assumed parallel to the long axis of GNR. Throughout the paper, the time factor exp(*−iωt*) is omitted.

We assume that the GNR is placed on a glass substrate in air. The effective refractive index of the surrounding medium is denoted by n; n = (1−*β*) n_sub_ + *β* n_air_, where the value of *β* is taken as 0.5, hence n = 1.25. The permittivity of gold is referred in [23]. Note that the wavelength of light, *λ,* throughout this paper is referred to that in vacuum; the corresponding wavelength in the surrounding medium is then *λ*/n. We employed the multiple multipole (MMP) method to analyze the electromagnetic field of the problem, based on the Maxwell’s equations [24,25].

The radiative power of bi-dipole is defined as

(1) Pr=12Re∫SE×H¯·da,

where *S* can be any arbitrary closed surface enclosing the GNR and the bi-dipole [25]. The nonradiative power due to the ohmic loss in GNR is defined as

(2) Pnr=−12Re∫ScE×H¯·da,

where *S*_c_ is the surface of GNR [25,26].

On the other hand, the dispersion relation of an infinitely long GNW can be obtained by solving the transcendental equation [27-29]

(3) $${ɛ}_{2}\zeta_{1}J_{1}\left(\zeta_{2}a\right)H_{0}^{\left(1\right)}\left(\zeta_{1}a\right)={ɛ}_{1}\zeta_{2}J_{0}\left(\zeta_{2}a\right)H_{1}^{\left(1\right)}\left(\zeta_{1}a\right)$$

where *J*_0_ and *J*_1_ are Bessel functions of the first kind of order 0 and 1, respectively, and *H*_0_^(1)^ and *H*_1_^(1)^ are Hankel functions of the first kind of order 0 and 1. Here, *ζ*_1_ and *ζ*_2_ are related to the wavenumber *k* as $\zeta_{i}^{2}=\mu \varepsilon_{i}\omega^{2}-k^{2}$, where *ε*_1_ and *ε*_2_ are the permittivity of the surrounding medium and gold, respectively, and *μ* is the permeability. The complex roots $k=k^{\prime }+ik^{\prime \prime }$ are found numerically to satisfy Equation 3 under the conditions, $k^{\prime }\geq 0$ and ω=2πc/λ, for a given angular frequency ω=2πc/λ, where *c* is the light speed in vacuum. The phase velocity of the surface plasmon wave in GNW is $v_{\text{p}}=\omega /k'$, and the group velocity $v_{\text{g}}=\partial \omega /\partial k^{\prime }$.

## Results and discussion

**Figure 2:**
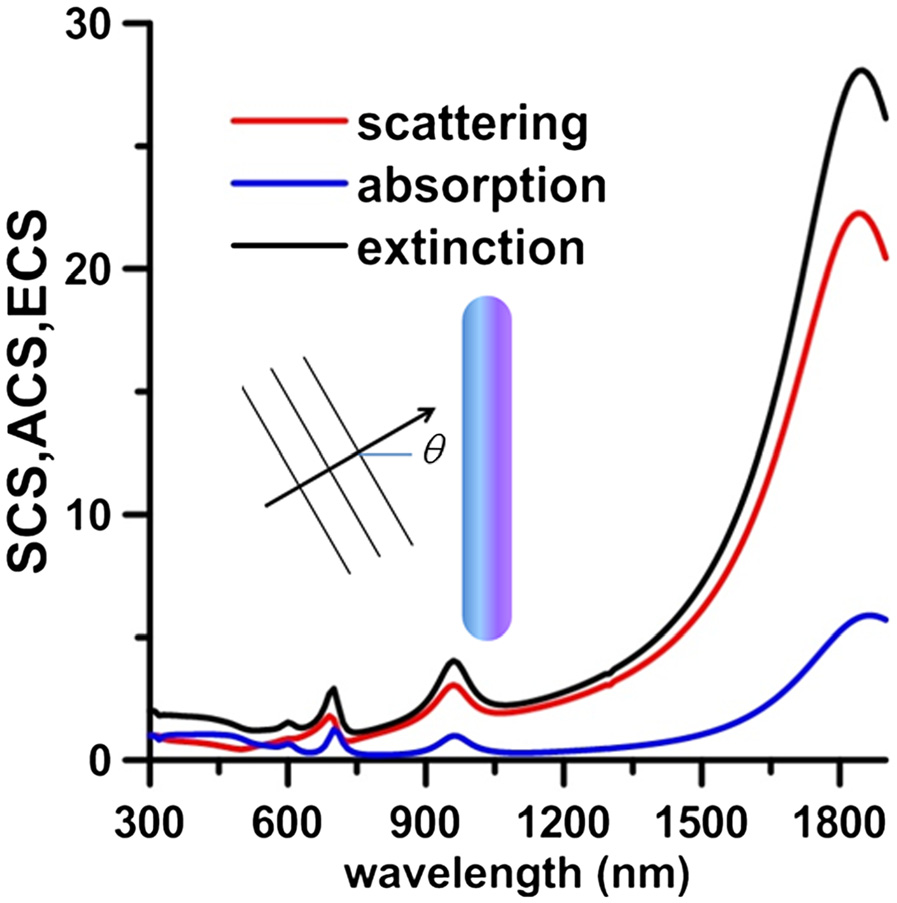
**SCS, ACS, and ECS efficiencies of GNR (*a*** **= 30 nm, AR = 8) irradiated by an obliquely incident plane wave.** SCS, ACS, and ECS efficiencies of GNR (*a* = 30 nm, AR = 8) irradiated by an obliquely incident plane wave of *θ* = 15°. The first, second, and third plasmon modes are at 1,870, 960, and 700 nm.

**Figure 3:**
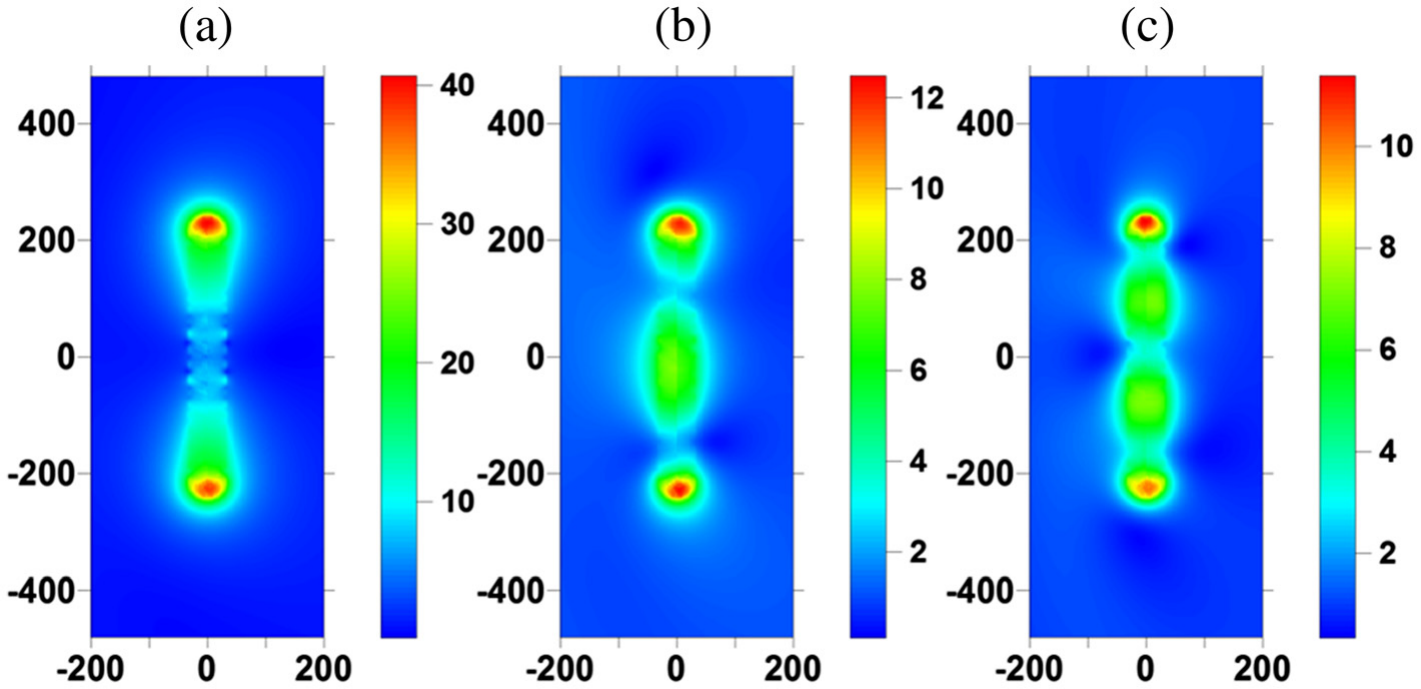
**Electric field distributions at the first, second, and third modes of GNR.** Electric field distributions at the (**a**) first (dipole, 1,870 nm), (**b**) second (quadrupole, 960 nm) and (**c**) third (sextupole, 700 nm) modes of GNR (*a* = 30 nm, AR = 8) irradiated by an obliquely incident plane wave of *θ* = 15°.

In order to identify the longitudinal plasmon modes of GNR, the optical responses of GNR is analyzed first. The scattering cross section (SCS), absorption cross section (ACS), and extinction cross section (ECS) efficiencies of GNR (*a* = 30 nm, AR = 8) irradiated by an obliquely incident plane wave of *θ* = 15° are shown in Figure 2. Here, the wavenumber vector and the polarization vector of the incident wave and the long axis of GNR are assumed to be in the same plane. These curves show that the first (dipole), second (quadrupole), and third (sextupole) plasmon modes are at 1,870, 960, and 700 nm, respectively. The normalized electric field distributions at these modes are also plotted in Figure 3. These results show that the two apexes of GNR are the hotspots at these longitudinal plasmon modes; the local electric field at these two apexes is amplified. This phenomenon is due to not only the lightening rod effect of GNR but also the strong plasmonic oscillation and implies that a femtosecond laser may excite two QDs located at the two apexes simultaneously through these plasmon modes if the excitation band of QDs coincides with one of these modes. As a result, the coherent emission of the two excited QDs could happen subsequently. Therefore, the coherent emission of the two QDs under the influence of GNR is worth of further study. In the following analysis, the two emitting QDs are modeled as two electric dipoles (bi-dipole) and the two-level emitters.

**Figure 4:**
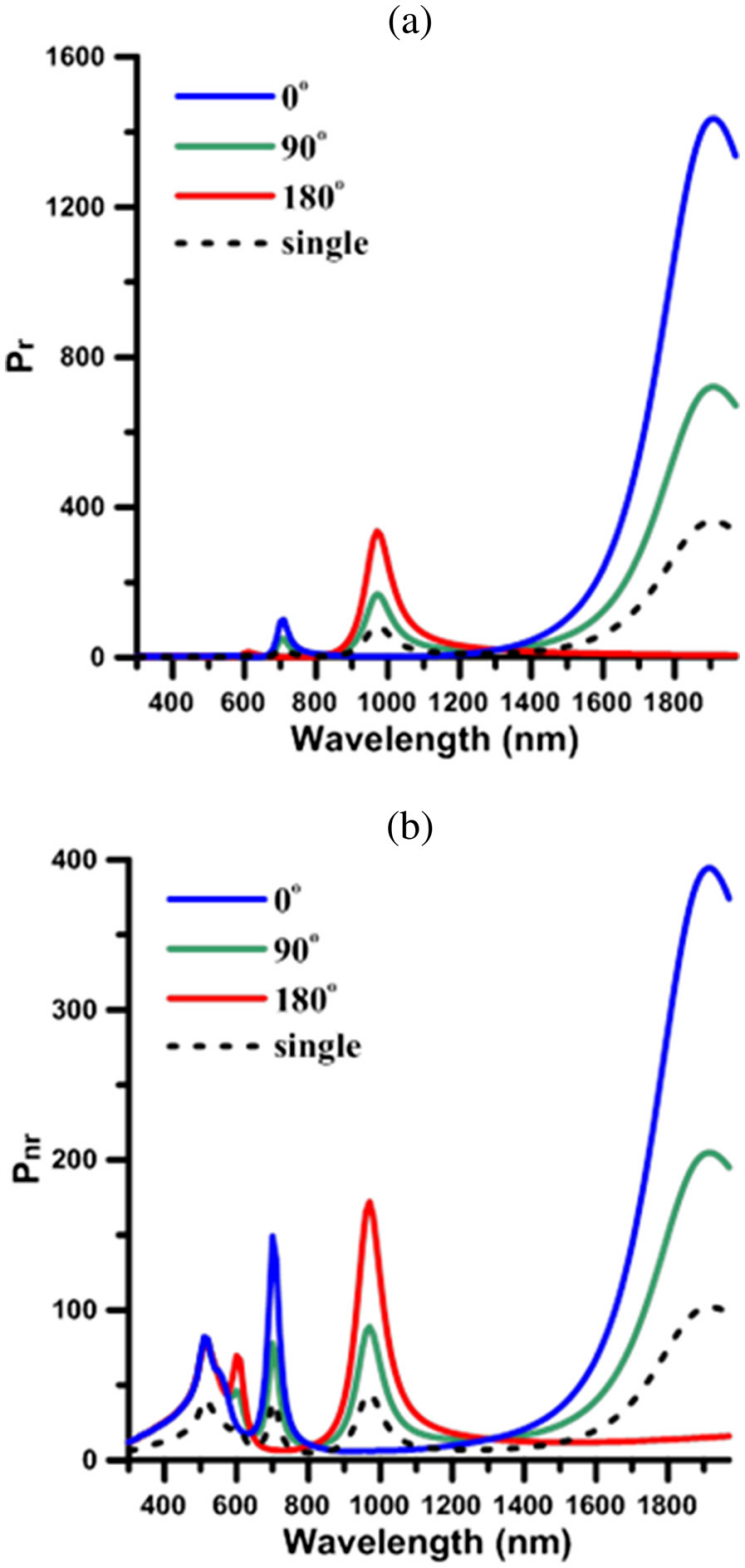
**Radiative power and nonradiative power of bi-dipole with phase difference.** (**a**) Radiative power and (**b**) nonradiative power of bi-dipole with phase difference (*φ* = 0°, 90°, 180°), *a* = 30 nm, AR = 8, *d* = 15 nm. Dashed line, single dipole.

Subsequently, the radiative and nonradiative powers of bi-dipole with phase difference (*φ* = 0°, 90°, 180°) in the presence of a GNR (*a* = 30 nm, AR = 8) are shown in Figure 4a,b respectively, where *d* = 15 nm. For comparison, the results for a single dipole in the presence of GNR are also presented. In this paper, the radiative and nanoradiative powers are normalized by the values of a free dipole’s radiative power. Figure 4a,b indicates that the first, second, third, and fourth modes are at 1,910, 970, 710, and 610 nm, respectively. The peaks of these plasmon modes are little red-shifted from those induced by a plane wave, where the first and second modes are at the NIR regime. Note that the absorption band with a peak at 520 nm, as shown in Figure 4b, is due to the interband transition in gold, rather than any longitudinal plasmon mode. Figure 4a,b suggests that the odd modes are easily induced by the in-phase (*φ* = 0°) bi-dipole due to the anti-symmetric configuration, but completely suppressed by the 180° out-of-phase bi-dipole. On the contrary, the even modes are easily induced by the 180° out-of phase bi-dipole due to the symmetric configuration, but suppressed by the in-phase one. For the case of *φ* = 90°, all the odd and even modes are induced, but the corresponding radiative and nonradiative powers are in between those for the cases of *φ* = 0° and *φ* = 180° bi-dipoles. Moreover, the radiative and nonradiative powers of bi-dipole interacting with GNR at these induced odd/even modes for cases of *φ* = 0/180° are nearly four times the corresponding values for the cases of a single dipole. In addition, because the radiative powers at the first and second modes dominate over the nonradiative ones, they belong to the bright modes at the far field. In contrast, the nonradiative powers at the third and fourth modes dominate over the radiative ones. This implies that these two higher-order modes are the dark modes. Through all of these plasmon modes, GNR exhibits the strong wavelength selectivity for the emission of emitters. The enhanced radiation and nonradiation occur only when the emission spectrum of QD overlaps with the specific plasmon mode of GNR. Compared to the other phase difference, the strongest emissions are induced by the in-phase bi-dipole coupled to the odd modes and the 180° out-of-phase bi-dipole to the even modes, respectively.

**Figure 5:**
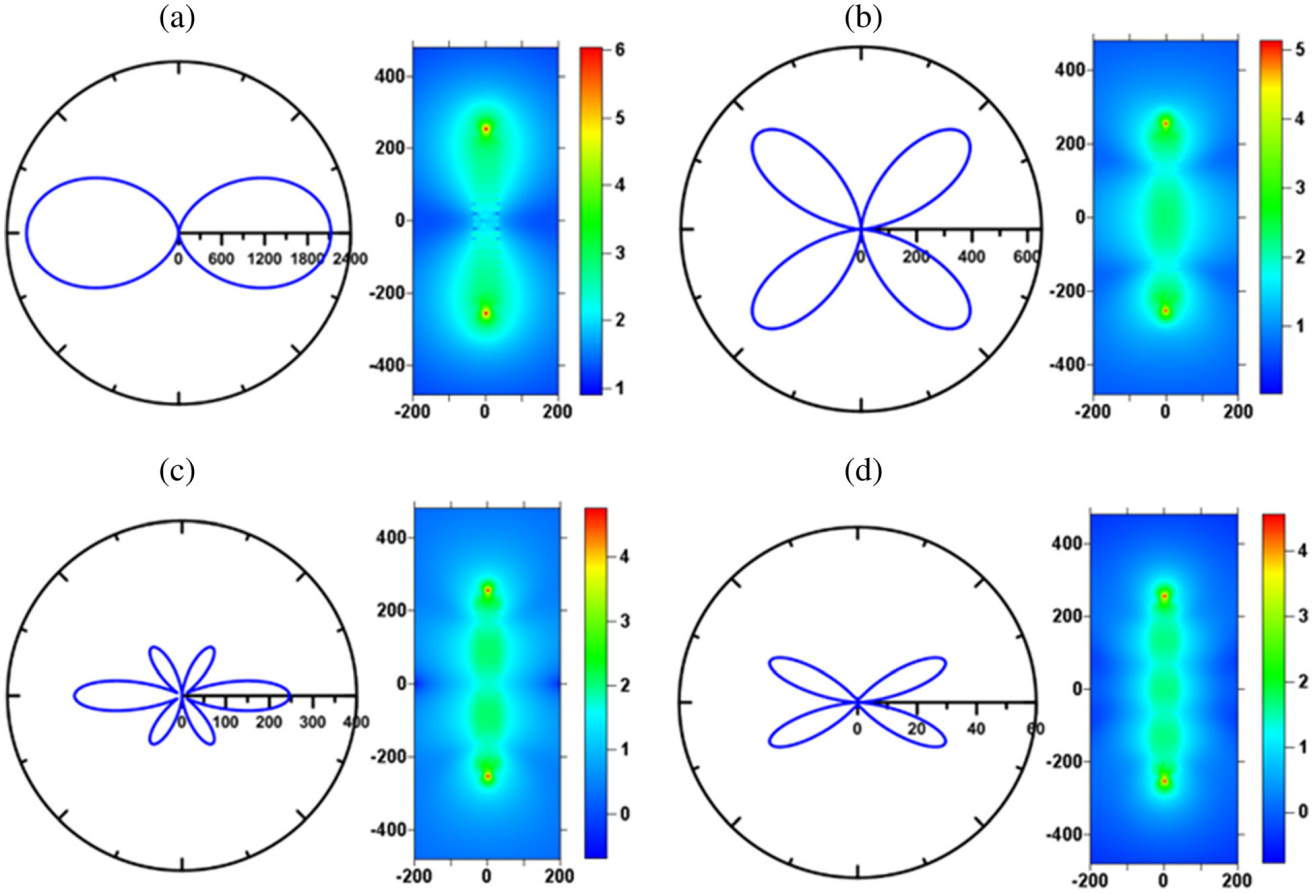
**Radiation patterns and near-field distributions of electric field of GNR (*a *= 30 nm, AR = 8).** (**a**) First (*λ*_1_ = 1,910 nm), (**b**) second (*λ*_2_ = 970 nm), (**c**) third (*λ*_3_ = 710 nm), and (**d**) fourth (*λ*_4_ = 610 nm) modes, where n = 1.25, *d* = 15 nm; *φ* = 0° for (a) and (c); *φ* = 180° for (b) and (d). Color bars are in logarithm scale.

The far-field radiation patterns, $\left|E\times \bar{H}\cdot e_{r}\right|$ for r>>λ versus angle, of the in-phase bi-dipole at the first and third surface plasmon resonance modes and 180° out-of-phase bi-dipole at the second and fourth plasmon modes are shown in Figure 5a,c,b,d, respectively. The corresponding electric near-field distributions are also shown for each mode, where the color bars are in logarithm scale. Note these radiation patterns are normalized by the maximum of the radiation pattern of a free dipole at the same *r*. From the radiation patterns, it is obvious that the first, second, third, and fourth modes of GNR correspond to the dipole, quadrupole, sextupole, and octupole modes, respectively. In particular, the pattern for the octupole mode is degenerated to have only four lobes, rather than eight, due to the ohmic loss in the GNR. Our results fully agree with [16]. Moreover, the distributions of the electric near-field at each mode, particularly the relative positions of nodal points, are consistent with the results of silver nanowire measured by EELS [19,20].

**Figure 6:**
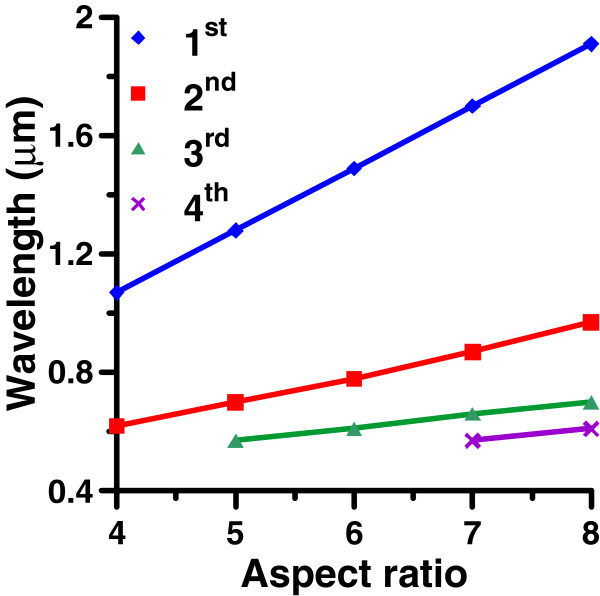
**Wavelengths of the first, second, third and fourth modes of GNR.** Wavelengths of the first, second, third, and fourth modes of GNR (*a* = 30 nm) versus AR (4 to 8) in a surrounding medium of n = 1.25.

Furthermore, the wavelengths of the first, second, third, and fourth modes of GNR (*a* = 30 nm) versus AR (4 to 8) for n = 1.25 are plotted in Figure 6, which illustrate that these modes are red-shifted as the AR of GNR increases. From the geometric viewpoint of nodal positions, the wavelength of the *m*-th mode resonant standing wave in the GNR can be given by $\lambda_{\text{sp}}=2L/m\text{.}$ We define a ratio, *α*

(4) $$\alpha =2\text{n}L/\left(m{\text{λ}}_{m}\right)\text{.}$$

**Figure 7:**
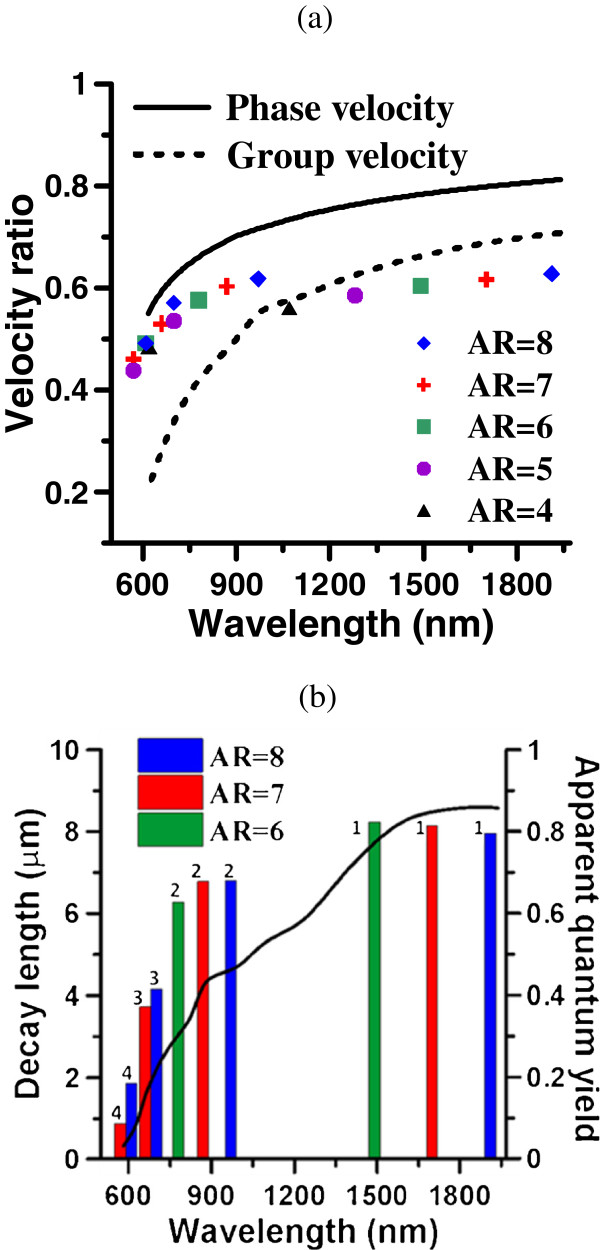
**Dispersion relation of GNW and decay length of GNW versus wavelength.** (**a**) Dispersion relation of GNW (*a* = 30 nm) in medium of n = 1.25, *d* = 15 nm. Solid line: phase velocity, dashed line: group velocity, and symbols: the plasmon modes of GNR. (**b**) Decay length (line) of GNW and apparent quantum yield (bar) of GNR (AR = 6, 7, 8) versus wavelength.

Here *m* denotes the *m*-th mode of GNR, and *λ*_*m*_ is the corresponding wavelength in vacuum of the peak in the radiative and nonradiative spectra. The value *α* is then related to the velocity ratio of the surface plasmon wave in GNR to the light speed in the medium. In addition, the dispersion relations of n*v*_p_/*c* and n*v*_g_/*c* of GNW (*a* = 30 nm) versus wavelength are plotted in Figure 7a according to Equation 3. The *α* value of each longitudinal mode of GNR with different ARs (4 to 8) is also plotted for comparison. The results show that these modes of GNR exhibit a correlation to the dispersion relation of GNW. In addition, the decay length $\left(1/k^{\prime \prime }\right)$ of GNW and the apparent quantum yield, $\eta =P_{\text{r}}/(P_{\text{r}}+P_{nr})$, of GNR (AR = 6, 7, 8) versus wavelength are shown in Figure 7b which illustrates that the tendency of the apparent quantum yields at these longitudinal modes of GNR are consistent with that of the decay length of GNW against the wavelength; the longer the wavelength, the larger the decay length and the apparent quantum yield are.

**Figure 8:**
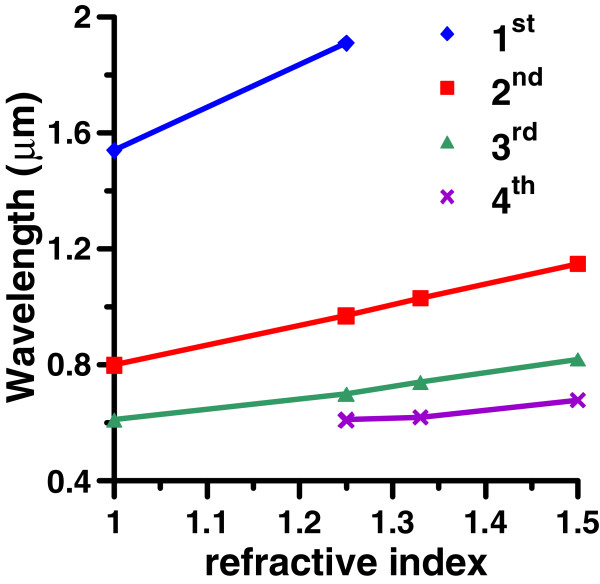
**Wavelengths of the first, second, third and fourth modes of GNR versus refractive index.** Wavelengths of the first, second, third and fourth modes of GNR (*a* = 30 nm) versus refractive index (n = 1 to 1.5) of medium for AR = 8.

Moreover, the effect of the surrounding medium on these plasmon modes is studied. The wavelengths of the first, second, third, and fourth modes of GNR (*a* = 30 nm) versus the refractive index (n = 1 to 1.5) of the surrounding medium for AR = 8 are shown in Figure 8. It is obvious that these modes are red-shifted as the refractive index of surrounding medium increases.

## Conclusions

Our analysis shows that the two apexes of GNR are hotspots, as an elongated GNR is irradiated by a plane wave at the plasmon modes. The phenomenon can increase the probability of the simultaneous excitation of a pair of QDs at these apexes. Consequently, the coherent emission of the two excited QDs may occur subsequently. They were modeled as two emitters: bi-dipole with phase difference. The radiative power of the bi-dipole at the apexes of the GNR shows the efficient nanoantenna effect for the emission of QDs at the first and second longitudinal plasmon modes which correspond to the dipole and quadrupole modes. Because the first and second modes of an elongated GNR are in the NIR regime, these modes can be used for the optical communication. On the other hand, the higher-order modes (e.g. the third and fourth modes) of GNR show the dark-mode behavior. Moreover, the odd modes are easily induced by the in-phase bi-dipole, but fully suppressed by the 180° out-of-phase one. On the contrary, the even modes are induced by the 180° out-of-phase bi-dipole, but suppressed by the in-phase one. Moreover, the strongest emissions are induced by the in-phase bi-dipole coupled to the odd modes, and the 180° out-of-phase one to the even modes, respectively. Summarily, the plasmon modes of GNR can enhance the simultaneous excitation and coherent emission of a pair of QDs.

These longitudinal plasmon modes of GNR are tunable by adjusting the AR as well as the permittivity of the surrounding medium. In addition, these modes of GNR are consistent with the dispersion relation of GNW. Our preliminary study shows the possibility of using an elongated GNR associated with two QDs at the ends as a subwavelength Fabry-Perot resonator [10] and might provide further insights for the nanorod spaser [16,30] and quantum optics [2,3]. Our analysis could be useful for the plasmonic applications in a variety of rapidly growing fields, e.g., surface enhanced fluorescence [25,26,31-33].

## Abbreviations

ACS: absorption cross section; AR: aspect ratio; ECS: extinction cross section; EELS: electric energy loss spectroscopy; GNR: gold nanorod; GNW: gold nanowire; MMP: multiple multipole; NIR: near-infrared; QD: quantum dot; SCS: scattering cross section.

## Competing interests

The authors declare that they have no competing interests.

## Authors’ contributions

JWL calculated dispersion relation of GNW and drafted the manuscript. CHH and BRC calculated EM field using MMP method and plotted the figures. MKK developed the MMP code, revised the manuscript, and approved the final version. All authors read and approved the final manuscript.
